# Supplementary material for: Nursing activities and associated workload of nurses in virtual care centres: A multicentre observational study
Source: PLOS Digit Health. 2025 Aug 12;4(8):e0000974. doi: 10.1371/journal.pdig.0000974 (PMC12342328; doi:10.1371/journal.pdig.0000974)
Supplement: S1 Appendix — (DOCX) [file pdig.0000974.s001.docx]

**S1 Appendix: survey used in the study**

*PART 1*

Q1. What is your position?

- Nurse
- Other, namely

Q2. What is your age? (years)

Q3. What is your gender?

- Male
- Female
- Other

Q4. What is the highest level of education you have completed?

- Secondary vocational education (Mbo)
- In-service trained
- Higher vocational education (Hbo)
- Higher vocational education (Hbo) advanced training
- University education (Wo) Bachelor's
- University education (Wo) Master's
- PhD
- Other, namely __________________________________________________

Q5. Hospital: In which hospital are you currently employed?

Q6. Years in healthcare: How many years have you been working in healthcare (including remote care)? Answers can be given in half years (e.g., 3.5 years).

Q7. Years in Virtual Care Center: How many years have you been working in the Virtual Care Center? Answers can be given in half years (e.g., 0.5 years).

Q8. Hours per week: How many hours per week do you work on average?

*PART 2*

You have reached part 2. This part consists of 15 questions. 'Work load’ consists of the following 6 factors: 1) Mental Demand, 2) Physical Demand, 3) Temporal Demand, 4) Effort, 5) Frustration Level, and 6) Performance. These 6 factors must be compared with each other. For each comparison between two factors, indicate which factor has more impact on work load compared to the other. The definitions of the factors are described in the questions. Each comparison represents a scale with a factor on each side. The description above the circles indicates to what extent one factor is more important than the other. Choose the circle that best describes how much impact a factor has on work load compared to the other factor.

Q1-5 AHP Mental: How much impact does 'Mental Demand’ have on experiencing work load compared to the other five factors? Choose an answer for each comparison.

*Mental Demand: Mental and perceptual activities (e.g., thinking, deciding, calculating, remembering, looking, and/or searching).*

*Physical Demand: Physical and bodily activities (e.g., pushing, pulling, turning, controlled, and/or active).*

*Temporal Demand: Time pressure related to the speed and pace at which something must be done.*

*Effort: Effort and exertion required to achieve/maintain a desired performance level.*

*Frustration: Degree of uncertainty, discouragement, irritation, and/or stress.*

*Performance: Degree of shortcomings with which you have achieved goals or activities and dissatisfaction with the extent of these shortcomings.*

|  | Extremely high impact | Very high impact | High impact | Moderate impact | Equal impact | Moderate impact | High impact | Very high impact | Extremely high impact |  |
| --- | --- | --- | --- | --- | --- | --- | --- | --- | --- | --- |
| Mental Demand | o | o | o | o | o | o | o | o | o | Physical Demand |
| Mental Demand | o | o | o | o | o | o | o | o | o | Temporal Demand |
| Mental Demand | o | o | o | o | o | o | o | o | o | Effort |
| Mental Demand | o | o | o | o | o | o | o | o | o | Frustration |
| Mental Demand | o | o | o | o | o | o | o | o | o | Performance |

Q6-9 AHP Physical: How much impact does 'Physical Demand’ have on experiencing work load compared to the other factors? Choose an answer for each comparison.

*Mental Demand: Mental and perceptual activities (e.g., thinking, deciding, calculating, remembering, looking, and/or searching).*

*Physical Demand: Physical and bodily activities (e.g., pushing, pulling, turning, controlled, and/or active).*

*Temporal Demand: Time pressure related to the speed and pace at which something must be done.*

*Effort: Effort and exertion required to achieve/maintain a desired performance level.*

*Frustration: Degree of uncertainty, discouragement, irritation, and/or stress.*

*Performance: Degree of shortcomings with which you have achieved goals or activities and dissatisfaction with the extent of these shortcomings.*

|  | Extremely high impact | Very high impact | High impact | Moderate impact | Equal impact | Moderate impact | High impact | Very high impact | Extremely high impact |  |
| --- | --- | --- | --- | --- | --- | --- | --- | --- | --- | --- |
| Physical Demand | o | o | o | o | o | o | o | o | o | Temporal Demand |
| Physical Demand | o | o | o | o | o | o | o | o | o | Effort |
| Physical Demand | o | o | o | o | o | o | o | o | o | Frustration |
| Physical Demand | o | o | o | o | o | o | o | o | o | Performance |

Q10-12 AHP Temporal Demand: How much impact does ‘Temporal Demand’ have on experiencing work load compared to the other factors? Choose an answer for each comparison.

*Temporal Demand: Time pressure related to the speed and pace at which something must be done.*

*Effort: Effort and exertion required to achieve/maintain a desired performance level.*

*Frustration: Degree of uncertainty, discouragement, irritation, and/or stress.*

*Performance: Degree of shortcomings with which you have achieved goals or activities and dissatisfaction with the extent of these shortcomings.*

|  | Extremely high impact | Very high impact | High impact | Moderate impact | Equal impact | Moderate impact | High impact | Very high impact | Extremely high impact |  |
| --- | --- | --- | --- | --- | --- | --- | --- | --- | --- | --- |
| Temporal Demand | o | o | o | o | o | o | o | o | o | Effort |
| Temporal Demand | o | o | o | o | o | o | o | o | o | Frustration |
| Temporal Demand | o | o | o | o | o | o | o | o | o | Performance |

Q13-14 AHP Effort: How much impact does 'Effort' have on experiencing work load compared to the other factors? Choose an answer for each comparison.

*Effort: Effort and exertion required to achieve/maintain a desired performance level.*

*Frustration: Degree of uncertainty, discouragement, irritation, and/or stress.*

*Performance: Degree of shortcomings with which you have achieved goals or activities and dissatisfaction with the extent of these shortcomings.*

|  | Extremely high impact | Very high impact | High impact | Moderate impact | Equal impact | Moderate impact | High impact | Very high impact | Extremely high impact |  |
| --- | --- | --- | --- | --- | --- | --- | --- | --- | --- | --- |
| Effort | o | o | o | o | o | o | o | o | o | Frustration |
| Effort | o | o | o | o | o | o | o | o | o | Performance |

Q15 AHP Frustration: How much impact does 'Frustration' have on experiencing work load compared to 'Performance'?

*Frustration: Degree of uncertainty, discouragement, irritation, and/or stress.*

*Performance: Degree of shortcomings with which you have achieved goals or activities and dissatisfaction with the extent of these shortcomings.*

|  | Extremely high impact | Very high impact | High impact | Moderate impact | Equal impact | Moderate impact | High impact | Very high impact | Extremely high impact |  |
| --- | --- | --- | --- | --- | --- | --- | --- | --- | --- | --- |
| Frustration | o | o | o | o | o | o | o | o | o | Performance |

PART 3

You have reached the third and final part of the questionnaire. A total of 5 topics are covered, divided into different questions. First, you indicate which activities you perform as a virtual care nurse. Then you assess the activities you perform based on the work load factors.

Q1 Training: Do you perform the following activities during your work? (Yes/no)

1. Organise and develop education and training for new care pathways or for introduction of new colleagues
2. Attending education and training to maintain and expand knowledge

Q2 Care Pathways: Do you perform the following activities during your work?

1. Develop protocols (work process and used technology) of new digital care pathways
2. Testing, evaluation, optimisation, and quality assurance of protocols (work process and technology used)
3. Active participation in quality assurance systems to improve the quality of care (e.g., VIM committee, quality monitoring and/or improvement of protocols and/or digital platforms)
4. Ambassadorship of virtual care (Communication towards external stakeholders to promote digital care).

Q3 Patient Contact: Do you perform the following activities during your work?

- 1. Planned remote patient counselling and coaching (e.g., for self-management, increased adherence, and/or psychological support.)
  2. Ad-hoc remote patient counselling and coaching (e.g., for self-management, increased adherence, and/or psychological support.)
  3. Planned remote communication with the patient to enable clinical decision-making (e.g., to verify data (e.g., measurement value) with the patient and/or give instructions on technology/instrument use).
  4. Ad-hoc remote communication with the patient to enable clinical decision-making (e.g., for verification of data (e.g., measurement value) with the patient and/or give instructions on technology/instrument use).

1. Performing nursing procedures (e.g., IV puncturing).

Q5 Administration: Do you perform the following activities during your work?

1. Keeping records of the care provided (e.g., in Luscii, Curavista, SanaNet, HiX).
2. Performing Human Resource Management (HRM) activities (e.g., schedules, HR administration).

You have indicated which activities you perform during your work. Now, assess how much work load you experience per activity based on the 6 work load factors. Use the numbers 0 to 100 to indicate how strongly a factor contributes to the experience of work load. Give a score of 0 if a factor does not contribute to work load. Give a score of 100 if a factor contributes maximally to work load.

For each question, the definitions of the workload variables are displayed as follows:

*Mental Demand: Mental and perceptual activities (e.g., thinking, deciding, calculating, remembering, looking, and/or searching).*

*Physical Demand: Physical and bodily activities (e.g., pushing, pulling, turning, controlled, and/or active).*

*Temporal Demand: Time pressure related to the speed and pace at which something must be done.*

*Effort: Effort and exertion required to achieve/maintain a desired performance level.*

*Frustration: Degree of uncertainty, discouragement, irritation, and/or stress.*

*Performance: Degree of shortcomings with which you have achieved goals or activities and dissatisfaction with the extent of these shortcomings.*

The following questions are answered using the sliders in the figure below. For every question, the six sliders are displayed to assess the activities.


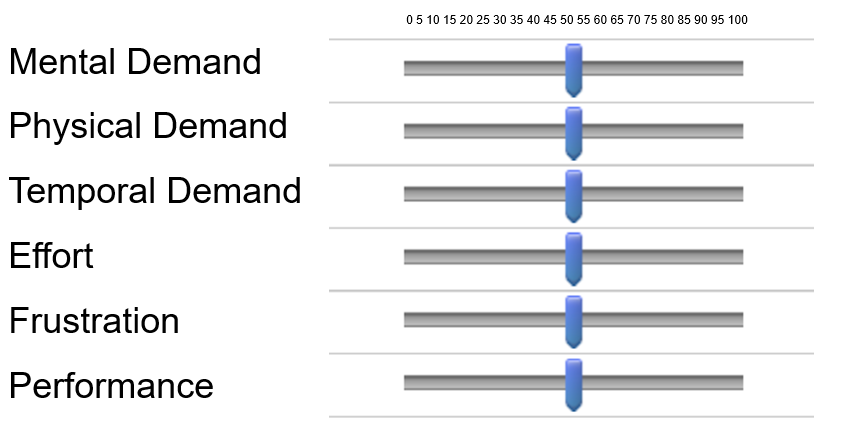


Q1 Training: Do you perform the following activities during your work? (Yes/no)

1. Organise and develop education and training for new care pathways or for introduction of new colleagues
2. Attending education and training to maintain and expand knowledge

Q2 Care Pathways: Do you perform the following activities during your work?

1. Develop protocols (work process and used technology) of new digital care pathways
2. Testing, evaluation, optimisation, and quality assurance of protocols (work process and technology used)
3. Active participation in quality assurance systems to improve the quality of care (e.g., VIM committee, quality monitoring and/or improvement of protocols and/or digital platforms)
4. Ambassadorship of virtual care (Communication towards external stakeholders to promote digital care).

Q3 Patient Contact: Do you perform the following activities during your work?

- 1. Planned remote patient counselling and coaching (e.g., for self-management, increased adherence, and/or psychological support.)
  2. Ad-hoc remote patient counselling and coaching (e.g., for self-management, increased adherence, and/or psychological support.)
  3. Planned remote communication with the patient to enable clinical decision-making (e.g., to verify data (e.g., measurement value) with the patient and/or give instructions on technology/instrument use).
  4. Ad-hoc remote communication with the patient to enable clinical decision-making (e.g., for verification of data (e.g., measurement value) with the patient and/or give instructions on technology/instrument use).

1. Performing nursing procedures (e.g., IV puncturing).

Q5 Administration: Do you perform the following activities during your work?

1. Keeping records of the care provided (e.g., in Luscii, Curavista, SanaNet, HiX).
2. Performing Human Resource Management (HRM) activities (e.g., schedules, HR administration).

*Evaluation of the Questionnaire*

Are there any activities you perform as a remote healthcare worker that were not covered in the assessment of work load?

Yes, namely __________________________________________________

No

General Comments: Do you have any other additions or comments regarding the questionnaire in general?

Yes, namely: __________________________________________________

No
