# Supplementary material for: Nursing activities and associated workload of nurses in virtual care centres: A multicentre observational study
Source: PLOS Digit Health. 2025 Aug 12;4(8):e0000974. doi: 10.1371/journal.pdig.0000974 (PMC12342328; doi:10.1371/journal.pdig.0000974)
Supplement: S2 Appendix — (DOCX) [file pdig.0000974.s002.docx]

**S2 Appendix: calculation example for nursing activity-associated workload**

Based on the pairwise comparisons of the six workload dimensions, nurse A has the following importance weights:

mental demand: 0.4,

physical demand: 0.05,

temporal demand: 0.1,

effort: 0.2,

frustration: 0.2,

performance: 0.05.

For the activity *calling patients*, she gave the following scores to the extent of which the variables contribute to this activity:

mental demand 70,

physical demand: 5,

temporal demand: 20,

effort: 40,

frustration: 30,

performance: 40.

Then, the final activities-associated workload of *calling patients* for nurse A is 50.25, as derived from:

$$Workload=\sum_{d=1}^{6} {IW}_{d}*S_{d}=0.4*70+0.05*5+0.1*20+0.2*40+0.2*30+0.05*40=46.25.$$

|  | **Importance weights** |  | **Scores for 1 activity** |  | **Calculation** |
| --- | --- | --- | --- | --- | --- |
| **Mental demand** | 0.4 | x | 70 | **=** | **28** |
| **Physical demand** | 0.05 | x | 5 | **=** | **0.25** |
| **Temporal demand** | 0.1 | x | 20 | **=** | **2** |
| **Effort** | 0.2 | x | 40 | **=** | **8** |
| **Frustration** | 0.2 | x | 30 | **=** | **6** |
| **Performance** | 0.05 | x | 40 | **=** | **2** |
| *Total* |  |  |  |  | **46.25** |
