# Supplementary material for: Nursing activities and associated workload of nurses in virtual care centres: A multicentre observational study
Source: PLOS Digit Health. 2025 Aug 12;4(8):e0000974. doi: 10.1371/journal.pdig.0000974 (PMC12342328; doi:10.1371/journal.pdig.0000974)
Supplement: S3 Appendix — (DOCX) [file pdig.0000974.s003.docx]

**S3 Appendix: calculation of consistency ratio**

In the AHP, it is common to check the consistency ratio (CR) of the pair-wise comparisons, as provided in the guidelines by Saaty et al. The CR is calculated by dividing the consistency index (CI) by the random index (RI). When comparing 6 elements, in this case the workload dimensions, RI = 1.24. For each participant, the CI is calculated individually, based on the following formula:

$$CI=(\lambda_{max}-n)/(n-1)$$

Here, $n$ is the number of compared elements, and for the calculation of $\lambda_{max}$we refer to Mu and Pereyra-Rojas.

Mu E, Pereyra-Rojas M. Understanding the Analytic Hierarchy Process. In: Saaty TL,. Practical Decision Making. Springer; 2016. Available from: https://www.springerprofessional.de/en/understanding-the-analytic-hierarchy-process/10588300
