# Supplementary material for: Nursing activities and associated workload of nurses in virtual care centres: A multicentre observational study
Source: PLOS Digit Health. 2025 Aug 12;4(8):e0000974. doi: 10.1371/journal.pdig.0000974 (PMC12342328; doi:10.1371/journal.pdig.0000974)
Supplement: S1 Table — (DOCX) [file pdig.0000974.s004.docx]

**S1 Table: The nursing activities list per round**

**First round:**

| **Activities category** | **Activity description** |
| --- | --- |
| 1. Education and training | 1. Organise and develop education and training for new care pathways or for induction of new colleagues |
|  | 2. Attending education and/or training to maintain and expand knowledge |
| 2. Development and promotion of new care pathways | 1. Develop protocols (work process and used technology) of new digital care pathways |
|  | 2. Testing, evaluation, optimisation, and quality assurance of protocols (work process and technology used) |
|  | 3. Active participation in quality assurance systems to improve the quality of care (e.g., VIM committee, quality monitoring and/or improvement of protocols and/or digital platforms) |
|  | 4. Ambassadorship of virtual care (Communication towards external stakeholders to promote digital care). |
| 3. Contact with patients | 1.1. Planned remote patient counselling and coaching (e.g., for self-management, increased adherence, and/or psychological support.) |
|  | 1.2. Ad-hoc remote patient counselling and coaching (e.g., for self-management, increased adherence, and/or psychological support.) |
|  | 2.1. Planned remote communication with the patient to verify data |
|  | 2.2. Ad-hoc remote communication with the patient to verify data |
|  | 3. Performing nursing procedures (e.g., IV puncturing). |
| 4. Clinical decision making | 1. Assess information from telemonitoring only via data platforms (e.g., Luscii, Curavista, SanaNet, HiX). |
|  | 2. Assess information only via patient contact (e.g., phone call). |
|  | 3. Assessing information from data platforms as well as patient contact. |
|  | 4. Decision-making based on protocols. |
|  | 5. Decision-making based on consultation with a hospital colleague (e.g., doctor, nursing specialist, and/or protocol owner). |
|  | 6.1. Planned handling of notifications from the monitoring platform used. |
|  | - 1. Ad-hoc handling of notifications from the monitoring platform used.   7.Applying generalist knowledge across multiple types of care pathways |
| 5. Administration | 1. Record keeping of care performed (e.g., in Luscii, Curavista, SanaNet, HiX et al.) |

**Second and third round:**

| **Activities category** | **Activity description** |
| --- | --- |
| 1. Education and training | 1. Organise and develop education and training for new care pathways or for introduction of new colleagues |
|  | 2. Attending education and training to maintain and expand knowledge |
| 2. Development and promotion of new care pathways | 1. Develop protocols (work process and used technology) of new digital care pathways |
|  | 2. Testing, evaluation, optimisation, and quality assurance of protocols (work process and technology used) |
|  | 3. Active participation in quality assurance systems to improve the quality of care (e.g., VIM committee, quality monitoring and/or improvement of protocols and/or digital platforms) |
|  | 4. Ambassadorship of virtual care (Communication towards external stakeholders to promote digital care). |
| 3. Contact with patients | 1.1. Planned remote patient counselling and coaching (e.g., for self-management, increased adherence, and/or psychological support.) |
|  | 1.2. Ad-hoc remote patient counselling and coaching (e.g., for self-management, increased adherence, and/or psychological support.) |
|  | 2.1. Planned remote communication with the patient to enable clinical decision-making (e.g., to verify data (e.g., measurement value) with the patient and/or give instructions on technology/instrument use). |
|  | 2.2. Ad-hoc remote communication with the patient to enable clinical decision-making (e.g., for verification of data (e.g., measurement value) with the patient and/or give instructions on technology/instrument use). |
|  | 3. Performing nursing procedures (e.g., IV puncturing). |
| 4. Clinical decision making | 1. Assess information from telemonitoring only via data platforms (e.g., Luscii, Curavista, SanaNet, HiX). |
|  | 2. Assess information only via patient contact (e.g., phone call). |
|  | 3. Assessing information from data platforms as well as patient contact. |
|  | 4. Decision-making based on protocols. |
|  | 5. Decision-making based on consultation with a hospital colleague (e.g., doctor, nursing specialist, and/or protocol owner). |
|  | 6. Coordinate and realise specialised hospital-at-home care (e.g. OPAT) |
|  | 7.1. Planned handling of notifications from the monitoring platform used. |
|  | 7.2. Ad-hoc handling of notifications from the monitoring platform used. |
| 5. Administration | 1. Record keeping of care performed (e.g., in Luscii, Curavista, SanaNet, HiX et al.) |
|  | 2. Perform Human Resource Management (HRM) duties (e.g., scheduling, HR administration) |
