# Supplementary material for: Nursing activities and associated workload of nurses in virtual care centres: A multicentre observational study
Source: PLOS Digit Health. 2025 Aug 12;4(8):e0000974. doi: 10.1371/journal.pdig.0000974 (PMC12342328; doi:10.1371/journal.pdig.0000974)
Supplement: S2 Table — (DOCX) [file pdig.0000974.s005.docx]

**S2 Table: Wilcoxon-Mann-Whitney test on tasks**

| 5.2 | 0.028 | 0.093 | 0.018 | 0.208 | 0.866 | 0.043 | 0.463 | 0.091 | 0.398 | 0.249 | 0.593 | 0.345 | 0.753 | 0.715 | 0.500 | 0.237 | 0.593 | 0.889 | 0.753 | 0.866 |  |
| --- | --- | --- | --- | --- | --- | --- | --- | --- | --- | --- | --- | --- | --- | --- | --- | --- | --- | --- | --- | --- | --- |
| 5.1 | 0.010 | 0.088 | 0.001 | 0.013 | 0.075 | 0.059 | 0.196 | 0.034 | 0.301 | 0.088 | 0.499 | 0.234 | 0.600 | 0.776 | 0.796 | 0.438 | 0.398 | 0.796 | 0.826 |  |  |
| 4.7.2 | 0.075 | 0.245 | <0.001* | 0.084 | 0.507 | 0.008 | 0.101 | 0.013 | 0.198 | 0.047 | 0.091 | 0.496 | 0.695 | 0.807 | 0.925 | 0.683 | 0.110 | 0.133 |  |  |  |
| 4.7.1 | 0.088 | 0.363 | 0.002 | 0.156 | 0.158 | 0.008 | 0.233 | 0.140 | 0.363 | 0.064 | 0.028 | 0.691 | 0.422 | 0.955 | 0.501 | 0.955 | 0.214 |  |  |  |  |
| 4.6 | 0.401 | 0.401 | 0.012 | 0.208 | 0.327 | 0.043 | 0.735 | 0.327 | 0.374 | 0.767 | 0.310 | 0.484 | 0.500 | 0.208 | 0.161 | 0.674 |  |  |  |  |  |
| 4.5 | 0.002 | 0.379 | <0.001* | 0.044 | 0.397 | 0.386 | 0.363 | 0.427 | 0.397 | 0.198 | 0.176 | 0.427 | 0.116 | 0.470 | 0.507 |  |  |  |  |  |  |
| 4.4 | 0.015 | 0.098 | 0.001 | 0.030 | 0.109 | 0.007 | 0.173 | 0.078 | 0.650 | 0.100 | 0.091 | 0.334 | 0.374 | 0.701 |  |  |  |  |  |  |  |
| 4.3 | 0.022 | 0.125 | <0.001 | 0.053 | 0.249 | 0.005 | 0.209 | 0.028 | 0.221 | 0.011 | 0.612 | 0.019 | 0.169 |  |  |  |  |  |  |  |  |
| 4.2 | 0.008 | 0.048* | 0.002 | 0.016 | 0.182 | 0.017 | 0.155 | 0.060 | 0.131 | 0.075 | 0.225 | 0.117 |  |  |  |  |  |  |  |  |  |
| 4.1 | 0.056 | 0.918 | <0.001* | 0.196 | 0.730 | 0.037 | 0.972 | 0.594 | 0.925 | 0.221 | 0.735 |  |  |  |  |  |  |  |  |  |  |
| 3.3 | 0.398 | 0.917 | 0.028 | 0.237 | 0.735 | 0.249 | 0.091 | 0.612 | 0.176 | 0.499 |  |  |  |  |  |  |  |  |  |  |  |
| 3.2.2 | 0.496 | 0.691 | 0.002 | 0.363 | 0.397 | 0.508 | 0.701 | 0.333 | 0.074 |  |  |  |  |  |  |  |  |  |  |  |  |
| 3.2.1 | 0.125 | 0.334 | 0.001* | 0.427 | 0.778 | 0.015 | 0.861 | 0.182 |  |  |  |  |  |  |  |  |  |  |  |  |  |
| 3.1.2 | 0.134 | 0.501 | 0.002 | 0.408 | 0.778 | 0.005 | 0.221 |  |  |  |  |  |  |  |  |  |  |  |  |  |  |
| 3.1.1 | 0.078 | 0.496 | 0.001* | 0.256 | 0.778 | 0.086 |  |  |  |  |  |  |  |  |  |  |  |  |  |  |  |
| 2.4 | 0.859 | 0.169 | 0.041 | 0.374 | 0.721 |  |  |  |  |  |  |  |  |  |  |  |  |  |  |  |  |
| 2.3 | 0.427 | 0.363 | 0.002 | 0.036 |  |  |  |  |  |  |  |  |  |  |  |  |  |  |  |  |  |
| 2.2 | 0.744 | 0.062 | 0.049 |  |  |  |  |  |  |  |  |  |  |  |  |  |  |  |  |  |  |
| 2.1 | 0.028 | <0.001* |  |  |  |  |  |  |  |  |  |  |  |  |  |  |  |  |  |  |  |
| 1.2 | 0.022 |  |  |  |  |  |  |  |  |  |  |  |  |  |  |  |  |  |  |  |  |
| 1.1 |  |  |  |  |  |  |  |  |  |  |  |  |  |  |  |  |  |  |  |  |  |
|  | 1.1 | 1.2 | 2.1 | 2.2 | 2.3 | 2.4 | 3.1.1 | 3.1.2 | 3.2.1 | 3.2.2 | 3.3 | 4.1 | 4.2 | 4.3 | 4.4 | 4.5 | 4.6 | 4.7.1 | 4.7.2 | 5.1 | 5.2 |

** Significance level p≤0.0002****Red*** *= negative rank*  ***Green****= positive rank*
