# Supplementary material for: Nursing activities and associated workload of nurses in virtual care centres: A multicentre observational study
Source: PLOS Digit Health. 2025 Aug 12;4(8):e0000974. doi: 10.1371/journal.pdig.0000974 (PMC12342328; doi:10.1371/journal.pdig.0000974)
Supplement: S3 Table — (DOCX) [file pdig.0000974.s006.docx]

**S3 Table: Wilcoxon-Mann-Whitney test on workload ad-hoc and planned activities**

| **Pair** | **Z statistic** | **2-tailed significance** |
| --- | --- | --- |
| **Activity 3.1.1– 3.1.2** | -1.223 | 0.221 |
| **Activity 3.2.1 – 3.2.2** | -1.784 | 0.074 |
| **Activity 4.7.1 – 4.7.2** | -1.503 | 0.133 |

** Significance level p ≤0.05*
