# Supplementary material for: Nursing activities and associated workload of nurses in virtual care centres: A multicentre observational study
Source: PLOS Digit Health. 2025 Aug 12;4(8):e0000974. doi: 10.1371/journal.pdig.0000974 (PMC12342328; doi:10.1371/journal.pdig.0000974)
Supplement: Attachment — Submitted filename: Review Comments to the Author (1).docx [file pdig.0000974.s008.docx]

**Review Comments to the Author**

**Reviewer #1**

Main Comments:

1. Clarity of Objectives: The rationale for using NASA-TLX and AHP together should be better explained. Why was this combination chosen, and how does it uniquely contribute to the

analysis?

We have expanded the explanation in the Methods section (step 2) to clarify the rationale for combining NASA-TLX and AHP as follows.

***Step 2: Workload definition and quantification***

*Workload is a concept that has been described in multiple ways. We chose a nurse-based subjective workload which is defined based on Hart et al.(19) definitions as the subjective effect on a nurse from performing activities. To measure subjective workload, we used the NASA-Task Load Index (TLX).(19) The NASA-TLX is a validated, employee-based questionnaire to measure subjective workload and is commonly used in studies in nursing.(20-23) The rationale of the NASA-TLX is that workload consists of six workload dimensions, that can be measured from two perspectives, the extent of which the workload dimension is present in a task, and the subjective weight of that dimension. E.g., if a task is physical very demanding, but a nurse does not perceive the physical demand dimension as an important part of workload, the subjective workload of this task for this dimension is still relatively low for this nurse.*

Page 8, Line 185-195

Additionally, we have discussed this approach extensively in the discussion section.

*Considering our method, combining the NASA-TLX with AHP is a novel approach, to the authors’ knowledge documented once in literature (20). This integration leverages the strengths of both tools to provide a comprehensive analysis of workload dimensions. The NASA-TLX is well-regarded for its ability to measure perceived workload across multiple dimensions, while AHP offers a structured technique for organizing and analysing complex decisions.(19,24,25) This combination allows for a more nuanced understanding of workload, particularly in complex environments like VCCs. In the conventional NASA-TLX, the weights are assigned based on ranking, meaning that dimensions cannot be valued equally, and that the lowest-ranked dimension is not included in the analysis.*

Page 19, Line 405-413

2. Sample Size and Generalizability: The sample size is small, and variability in VCC characteristics may limit the generalizability of findings. This limitation should be explicitly discussed in the manuscript.

We do agree the sample size is relatively small and VCC characteristics vary. We addressed this in the first paragraph of the limitations section. In line with the comment of Reviewer 3, we have expanded this section.

***Limitations***

*Several limitations should be addressed in the interpretation of our results. First, while the inclusion of six centres adds breadth to the study and there is no sight on the total population of VCC nurses in the Netherlands (which may be small given the novelty of VCCs), the overall sample remains limited (19 participants). The study was conducted in six VCCs with varying sizes, patient numbers, care pathways, and technological setups. This variability could affect the generalizability of the findings, as differences in technological infrastructure and patient populations may lead to distinct experiences and workloads for nurses across different centres. However, due to the dynamic and heterogeneous nature of VCCs, obtaining consistent and comprehensive data across all hospitals was not feasible. Future research should aim to collect and analyse such detailed data to facilitate in-depth comparison of VCC characteristics. Additionally, workload may be influenced by factors such as age, sex, or other contextual variables, which were not fully captured in this study due to the sample size constraints.(19,23)*

Page 19, Line 422-434

3. Workload Insights: Tasks such as protocol development were identified as most demanding but lack detailed discussion. Provide more granular insights into what makes these tasks challenging (e.g., technical complexity, coordination requirements).

To provide insights in what factors makes the tasks challenging was not the goal of our study. Nevertheless, we have enhanced our discussion section about these potential challenges of the tasks in relation to the workload.

*Also, we found VCC nurses have a pivotal role in the development and promotion of new care pathways. The development of protocols, which often include a substantial technological component, their practical testing, and continuous improvement seem to be integral parts of VCC nurses’ responsibilities. These tasks are particularly demanding due to their technical complexity, the need for precise coordination among multidisciplinary teams, and the continuous adaptation to new technologies. While these responsibilities are crucial for ensuring high-quality care, it remains uncertain whether nurses can perform these tasks effectively and whether they perceive them as appropriate within their scope of duties. This task causes the highest workload (medium) among all tasks, probably due to the aforementioned complexities. Therefore it seem to be important to support or educate nurses in this task to decrease workload, as high workload is associated with higher nursing burnout and retention.(23) Additionally, we noted that the nurses act as ambassadors in the implementation of virtual care, which aligns with current trends in nursing leadership and autonomy in implementation of novel (virtual) care pathways.(29,31) However, this is a relative novel role for nurses which also suggest additional education is needed for instance in project management or nurse leadership, or may advocate for task allocation within the nurses or to other disciplines.*

Page 18, Line 368-383

4. Ambassadorship and Education Roles: Reassess whether these tasks should fall within the scope of nursing roles or require specialized staff. This is an important point for broader discussions on task allocation.

We do agree this is a relevant point. Therefore, we did mention this in the implications section of the discussion but extended the discussion to this point.

***IMPLICATIONS***

*Although most nursing activities were associated with a low to medium workload, addressing the higher workload levels of VCC nurses’ activities is crucial for several reasons. One potential strategy is to implement task differentiation within VCCs. This approach can help gaining expertise in certain activities (i.e. developing new care protocols) and distribute the workload more evenly among staff, preventing burnout and enhancing overall efficiency.(37) Additionally, it is important to reassess whether certain tasks, such as protocol development and technological implementation, should fall within the scope of nursing roles or require specialized staff. This reassessment can ensure that nurses are not overburdened with tasks that may be outside their traditional scope of practice, thereby improving job satisfaction and retention. By clearly defining roles and responsibilities, VCCs can optimize task allocation and enhance the overall effectiveness of the care provided.*

Page 21, Line 463-473

5. Comparative Analysis: The manuscript claims that VCC work is less physically demanding than traditional nursing roles but lacks comparative data. Include evidence or references to support this claim.

In the Comparison with prior work, we discussed our findings with other studies using the NASA-TLX among nurses. We have made this comparison more explicit and enhanced the discussion.

*Furthermore, our study revealed that VCC nurses overall rated their workload as very low or low. Although the mental demand was also the highest workload factor for VCC nurses in comparison to prior studies, it seems this workload is generally lower than conventional and Intensive Care Unit nurses working in clinical care.(20) Specifically, it appears to be lower in physical demand.(21,22) This contrast is likely attributable to the nature of VCC work, which predominantly involves remote patient interactions and remote monitoring, requiring less physical exertion than traditional hands-on patient care.(21,23) Consequently, the reduced physical demands may explain the lower workload ratings by VCC nurses, highlighting a significant shift in the physical aspects of nursing tasks between these two environments. Conversely, the reduced physical activity associated with sedentary work may contribute to the development of back pain issues*.

Page 18, Line 357-368

6. Figures and Tables: Consider improving the visual representation of workload categories in Figure 2 for better comparison between activities.

We have added the workload categories to Figure 2 on page 15 to improve the visual representation.

Minor Comments:

7. Address grammatical and formatting inconsistencies.(e.g., "the highest associated workload is suggest the need"). Proofreading is necessary.

We have proofread the whole manuscript with the researchers and made corrections where necessary throughout the manuscript.

8. Strengthen the discussion of methodological limitations, particularly around the inconsistency in pairwise comparisons.

We have strengthened the discussion on this topic by extending the discussion of this limitation.

*Lastly, although the NASA-TLX and AHP provide a more valid method, they may also exaggerate extreme scores. Future studies should consider to apply this combined methodology in different healthcare settings to validate this method further and compare and contrast workload perceptions across various contexts. This would provide a broader perspective on the applicability and robustness of the NASA-TLX and AHP combination. Additionally, our study showed that only 7 (37%) of nurses were consistent in their pair-wise comparisons of the workload dimensions. This inconsistency should be taken into account when interpreting the results, as it suggeststhat consistently comparing workload dimensions is a complex task for nurses. The inconsistency in pairwise comparisons may be attributed to several factors, including the subjective nature of workload assessment, the cognitive load involved in making multiple comparisons, and potential variations in individual perceptions of workload dimensions. Future research should explore strategies to improve the consistency of pairwise comparisons, such as simplifying the comparison process or providing real-time feedback when filling in the survey.*

Page 20, Line 449-461

**Reviewer #2**

1. The study design is robust, using validated workload assessment tools (NASA-TLX, AHP). However, the workload measurement method could be explained more clearly for those unfamiliar with it.

Thank you for this feedback. We have clarified the workload measurement method in the methods section of our manuscript.

***Step 2: Workload definition and quantification***

*Workload is a concept that has been described in multiple ways. We chose a nurse-based subjective workload which is defined based on Hart et al.(19) definitions as the subjective effect on a nurse from performing activities. To measure subjective workload, we used the NASA-Task Load Index (TLX).(19) The NASA-TLX is a validated, employee-based questionnaire to measure subjective workload and is commonly used in studies in nursing.(20-23) The rationale of the NASA-TLX is that workload consists of six workload dimensions, that can be measured from two perspectives, the extent of which the workload dimension is present in a task, and the subjective weight of that dimension. E.g., if a task is physical very demanding, but a nurse does not perceive the physical demand dimension as an important part of workload, the subjective workload of this task for this dimension is still relatively low for this nurse.*

*The six workload dimensions are: mental demand, physical demand, temporal demand, effort, frustration level, and performance. Together, these variables represent the total workload of activities. The NASA-TLX assigns a quantitative workload measure to activities based on the weights of these six dimensions and the extent to which each dimension is present in each activity.*

*Weights were assigned to the workload dimensions through pairwise comparisons by means of the Analytical Hierarchy Process (AHP). Combining the NASA-TLX with the AHP provides more accurate weights in comparison with the conventional method within the NASA-TLX, as proposed by Virtanen et al.(24). The AHP provides the option to assess variables as equally important and therefore include all six variables in the workload, which is not possible in the conventional method.(24) The aim of these pairwise comparisons is to assign a numerical weighted importance to all six workload dimensions, per nurse, resulting in a sum of 1. To come to this, according to the AHP, participants compared two variables at a time to determine which was more important on a cardinal scale ranging from -9 to 9.(25) A total of 15 comparisons were made to establish the relative importance of the dimensions, which were then used to determine their weights. An example of the outcome could be mental demand: 0.4, physical demand: 0.05, temporal demand: 0.1, effort: 0.2, frustration: 0.2, performance: 0.05.*

Page 8, Line 185-212

2. Discussion provides meaningful interpretation but has some redundancy. The workload findings and technology-related insights are interesting but could be more critically analyzed.

We have removed the redundancy in our discussion and extended the workload findings in relation to prior research and technology-related insights.

*The two tasks of developing protocols (work process and used technology) of new digital care pathways and testing, evaluating, optimising, and assuring quality of protocols (work process and technology used) tasks seem to generate more workload (medium) in comparison to the other tasks where we found that they generate very low or low workload experienced by nurses. This could be because the other task categories (e.g. clinical decision making and patient contact) align with conventional nursing work and/or what they learned during their training. Especially because VCC nurses generally had relevant prior work experience in nursing before working in the VCC. This could have helped to gain experience and become familiar with these tasks categories. Despite this, the use of technology by the VCC nurses does not seem to cause a substantial high workload, which might be different for conventional nurses.(32,33) This could be because VCC nurses may naturally be more technology-minded than the majority of the nursing workforce, which is often slower to adopt new technologies and innovations.(34) The integration of technology in VCCs requires nurses to engage in complex tasks such as developing and optimizing digital care protocols, which involve significant technical complexity and coordination among multidisciplinary teams. These tasks demand a high level of technological proficiency and adaptability, which VCC nurses seem to possess to a certain extent. Moreover, the continuous adaptation to new technologies and the need for precise coordination highlight the importance of ongoing education and support for VCC nurses.*

Page 19, Line 384-401

**Reviewer #3**

Thank you for giving me the opportunity to review this manuscript. The paper is well and quite easy to follow. I had a serious problem with the sample size but the authors acknowledged that as a limitation. I have few suggestions to offer except the following

1. The data analysis part is quite short and that will be difficult for other researchers to replicate in other contexts. The authors should consider expanding on the data analysis approach, particularly the process in which they identified the nursing activities. If the nurses were consulted/interviewed, how did the authors analyzed that data to arrive at the nursing activities or how did the data inform the revisions of those nursing activities. These processes need to be made explicit.

To ensure the robustness and replicability of our study, we have expanded on the data collection and the data analysis approach, particularly the process of identifying nursing activities. (Page 7-Page 10)

We acknowledge that the data analysis section is brief and may pose challenges for replication in other contexts. In our study, we employed a three-step pragmatic approach, as outlined in the data collection section. The identification of nursing activities involved consulting with nurses and the research team. These consultations and interviews were analyzed through a systematic process of discussion for each task and oral agreement to reach consensus on the nursing activities. However, more rigorous data collection and analysis methods, such as a formal Delphi round, were not performed.

We have clarified this in the methods section and added the outcomes of each step in the Appendix S2. Additionally, we have included this limitation in the manuscript's limitations section.

***Step 1: Nursing activities***

*For the development of the activities list, three VCC nurses from Hospital A, who have extensive experience in providing remote care, were involved. These nurses were selected to develop a general and concise activities list that accurately represents the profession of a VCC nurse.*

*The activities list was developed through a three-round process. Initially, the research team (JL, JLV, CV) created a preliminary concept based on themes from a previous qualitative study on the work of VCC nurses and a study on nurses working with remote care in home care settings.(10) To account for activities that are approached either ad-hoc or in a planned manner, this differentiation was made in the activity descriptions, as previous studies indicated that unplanned activities might result in a higher perceived workload.(17,18) (S1 Table) In the second round, the activities list was discussed and verified by the three VCC nurses and JL, JLV and CV. The list was further refined using the seven Canadian Medical Education Directions for Specialists (CanMEDS) roles from the Dutch educational profile for bachelor nurses (Bachelor Nursing 2030). Finally, the list was discussed with the nurses again but no additions were made, and consensus was reached together with the research team based on verbal agreement. In the survey, nurses of the other hospitals were able to to indicate whether they performed each activity and could rate the activity as ‘not applicable’ if they did not perform the task.*

Page 7, Line 167-183

*Third, the activities list used in this study was developed through input from a small group of experienced VCC nurses of one hospital and refined via consensus. Although this hospital is one of the frontrunners in the field of VCCs, it may not encompass the full range of activities or account for variations in practice across different VCCs. However no missed activities in open text field mentioned by nurses from other five hospitals. Additionally, our approach to identifying nursing activities involved a three-step pragmatic method, including consultations and interviews with nurses and the research team. These consultations were analysed through a systematic process of discussion for each task and oral agreement to reach consensus. While this method provided valuable insights, it lacks the rigor of more formal data collection and analysis methods, such as a Delphi round.*

Page 20, Line 440-448

2. The authors talk of remote and virtual care and I am not sure if there any differences between the two

We have clarified the definitions in the introduction of the manuscript and adapted the terms of virtual to remote throughout the manuscript.

*One promising model is care delivery in a Virtual Care Centre (VCC). Although there is no standardized definition of a VCC, in this study we define VCCs as a hospital department that specialise in providing remote care to enable remote disease monitoring and facilitate interaction between patients and healthcare professionals without the need for an in-person visit or admission to a healthcare facility.(6,7) This remote care is virtual and delivered through various communication tools such as telephone and video calls, secure messaging platforms, and mobile applications for remote monitoring and connecting patients with healthcare professionals.(7,8)*

Page 5, Line 93-99

**Reviewer #4**

See comments/suggestions
Abstract

1. Double check for clarity: The highest associated workload is suggest the need...

We have double-checked the abstract for clarity and corrected any unclear statements

2. Indicate breakdown of 21 nursing activities for each area.

We have indicated the breakdown of 21 nursing activities for each area in the abstract.

Introduction

3. To better organize your results later, it is good to explicitly write your research objectives at the end of the introduction.

We have explicitly stated the research objectives at the end of the introduction.

*Therefore, this study aims to 1) identify the nursing activities performed in VCCs and 2) assess nurses’ perceived workload associated with these activities.*

Page 6, Line 129-131

Methods

4. Possible to create a table summarizing how different each VCCs are? Like a table comparing each VCC based on the aspects your mentioned.

Given the evolving nature of VCCs and the variability in their characteristics, it was not feasible to collect detailed data for each hospital. We have added this to our limitation section as this may be relevant to collect such data for a more granular comparison of VCC characteristics.

*However, due to the dynamic and heterogeneous nature of VCCs, obtaining consistent and comprehensive data across all hospitals was not feasible. Future research should aim to collect and analyse such detailed data to facilitate in-depth comparison of VCC characteristics.*

Page 20, Line 426-429

5. What do you mean by this statement: "nurses of the other hospitals were given the opportunity to indicate whether they performed each activity."?

We have clarified this text to make clear that in the survey, nurses from other hospitals had the opportunity to indicate whether they performed each activity and could also rate the activity as 'not applicable' if they did not perform the task.

*In the survey, nurses of the other hospitals were able to indicate whether they performed each activity and could rate the activity as ‘not applicable’ if they did not perform the task.*

Page 8, Line 181-183

6. Was consensus about activities reached through qualitative (verbal agreement) or quantitative means (computing interrater reliability?)

This was through verbal agreement. We have clarified this in the methods section on Page 8, Line 179-183

7. Need citations to justify that TLX is commonly used in nursing studies.

We have added the citations to justify this.

8. Need citations to support combining TLX with AHP. Has this been done before? Similar to the use of CR also. Also it is novel to do that (based on the discussion section), was there any inspiration from previous work to do this approach?

We have clarified in the text why we used a combination of TLX with AHP and the use of the CR. Furthermore, we have enhanced the novelty discussion.

*Weights were assigned to the workload dimensions through pairwise comparisons by means of the Analytical Hierarchy Process (AHP). Combining the NASA-TLX with the AHP provides more accurate weights in comparison with the conventional method within the NASA-TLX, as proposed by Virtanen et al.(24). The AHP provides the option to assess variables as equally important and therefore include all six variables in the workload, which is not possible in the conventional method.(24) The aim of these pairwise comparisons is to assign a numerical weighted importance to all six workload dimensions, per nurse, resulting in a sum of 1. To come to this, according to the AHP, participants compared two variables at a time to determine which was more important on a cardinal scale ranging from -9 to 9.(25) A total of 15 comparisons were made to establish the relative importance of the dimensions, which were then used to determine their weights. An example of the outcome could be mental demand: 0.4, physical demand: 0.05, temporal demand: 0.1, effort: 0.2, frustration: 0.2, performance: 0.05.*

Page 8, Line 200-212

*Considering our method, combining the NASA-TLX with AHP is a novel approach, to the authors’ knowledge documented once in literature (20). This integration leverages the strengths of both tools to provide a comprehensive analysis of workload dimensions. The NASA-TLX is well-regarded for its ability to measure perceived workload across multiple dimensions, while AHP offers a structured technique for organizing and analysing complex decisions.(19,24)(25) This combination allows for a more nuanced understanding of workload, particularly in complex environments like VCCs. In the conventional NASA-TLX, the weights are assigned based on ranking, meaning that dimensions cannot be valued equally, and that the lowest-ranked dimension is not included in the analysis. Although this combination provides a detailed analysis, it may also exaggerate extreme scores. For instance, VCC nurses report low physical demands in their work, leading to lower scores in that dimension. This should be carefully considered when interpreting the results, as it may skew the perceived workload in certain areas. Additionally, inconsistencies in participants’ responses indicate potential issues with the AHP's subjective pairwise comparisons. Despite these challenges, the combined use of NASA-TLX and AHP enhances the ability to identify specific workload components and their relative importance, which is beneficial for targeted interventions and improvement of nurses’ work.*

Page 19, Line 402-417

9. Include the survey form used in the study.

We have added the survey to the Appendix of the study.

Results

10. In table 2, why assign 1.1, 1.2 for some items instead of separate counts (1,2,3,4,5...)?

We remained this count of the tasks, as these tasks are similar to a certain extent and underline this, except for the ad-hoc or planned nature of the task."

11. For Figure 2, I feel that this needs to be sorted based on the most to lowest workload.

We have opted not to sort the tasks based on workload from highest to lowest because clustering by categories provides a more logical organization. This approach aligns with Reviewer 1, Comment 6, and we have made adjustments accordingly to clarify the categories in the revised Figure 2. Additionally, we believe this format allows readers the flexibility to determine the most appropriate sorting method based on median values or categories.

12. Can you clarify this?: "For n=7 (37%) nurses their CR-score was below the limit of 0.1, implying consistency"

We have extended the limitations section to clarify this.

*Additionally, our study showed that only 7 (37%) of nurses were consistent in their pair-wise comparisons of the workload dimensions. This inconsistency should be taken into account when interpreting the results, as it suggests that consistently comparing workload dimensions is a complex task for nurses. The inconsistency in pairwise comparisons may be attributed to several factors, including the subjective nature of workload assessment, the cognitive load involved in making multiple comparisons, and potential variations in individual perceptions of workload dimensions. Future research should explore strategies to improve the consistency of pairwise comparisons, such as simplifying the comparison process or providing real-time feedback when filling in the survey.*

Page 21, Line 453-461

13. Is it possible to compute a combined workload rating at the category level?

We have reported this in the Results-section

***Nursing activity associated workload***

*For the categories, median workload for education and training was 28.1 (IQR: 11.2–48.4), for development and promotion of new care pathways was 32.9 (IQR: 24.1–49.0), for contact with patients was 15.6 (IQR: 6.4–38.7), for clinical decision making was 14.8 (IQR: 6.0–21.4) and for administration was 8.9 (IQR: 7.3–24.1).*

Page 16, Line 310-314

Discussion

14. I think it is important to account for social desirability when the participants judge their workload. Perhaps a discussion on that can be made in the limitations section.

We have discussed the impact of social desirability on workload judgments in the limitations section.

*Although the survey was anonymized, socially desirable responses may have influenced the study results on perceived workload as nurses might have reported lower workload levels to align with perceived expectations or norms.*

Page 20, Line 437-439
